# Region-Dependent Increase of Cerebral Blood Flow During Electrically Induced Contraction of the Hindlimbs in Rats

**DOI:** 10.3389/fphys.2022.811118

**Published:** 2022-03-23

**Authors:** Remi Chaney, Philippe Garnier, Aurore Quirié, Alain Martin, Anne Prigent-Tessier, Christine Marie

**Affiliations:** ^1^ INSERM UMR1093-CAPS, Université Bourgogne Franche-Comté, UFR des Sciences de Santé, Dijon, France; ^2^ Département Génie Biologique, IUT, Dijon, France; ^3^ INSERM UMR1093-CAPS, Université Bourgogne Franche-Comté, UFR des Sciences du Sport, Dijon, France

**Keywords:** cerebral blood flow (CBF), heart rate (HR), exercise pressor reflex, rat—brain, electrically-induced muscle contraction

## Abstract

Elevation of cerebral blood flow (CBF) may contribute to the cerebral benefits of the regular practice of physical exercise. Surprisingly, while electrically induced contraction of a large muscular mass is a potential substitute for physical exercise to improve cognition, its effect on CBF remains to be investigated. Therefore, the present study investigated CBF in the cortical area representing the hindlimb, the hippocampus and the prefrontal cortex in the same anesthetized rats subjected to either acute (30 min) or chronic (30 min for 7 days) electrically induced bilateral hindlimb contraction. While CBF in the cortical area representing the hindlimb was assessed from both laser doppler flowmetry (LDF_CBF_) and changes in p-eNOS^Ser1177^ levels (p-eNOS_CBF_), CBF was evaluated only from changes in p-eNOS^Ser1177^ levels in the hippocampus and the prefrontal cortex. The contribution of increased cardiac output and increased neuronal activity to CBF changes were examined. Stimulation was associated with tachycardia and no change in arterial blood pressure. It increased LDF_CBF_ with a time- and intensity-dependent manner as well as p-eNOS_CBF_ in the area representing the hindlimb. By contrast, p-eNOS_CBF_ was unchanged in the two other regions. The augmentation of LDF_CBF_ was partially reduced by atenolol (a ß1 receptor antagonist) and not reproduced by the administration of dobutamine (a ß1 receptor agonist). Levels of c-fos as a marker of neuronal activation selectively increased in the area representing the hindlimb. In conclusion, electrically induced bilateral hindlimb contraction selectively increased CBF in the cortical area representing the stimulated muscles as a result of neuronal hyperactivity and increased cardiac output. The absence of CBF changes in cognition-related brain regions does not support flow-dependent neuroplasticity in the pro-cognitive effect of electrically induced contraction of a large muscular mass.

## Introduction

Induced skeletal muscle contraction (ISMC) has been shown to improve muscle strength (Duchateau and Hainaut, 1988) and functional capacity (Maddocks et al., 2016). Over the last few years, ISMC has broadened its field of action beyond the muscle. For instance, improvement of motor function by ISMC was related not only to muscular changes but also to induction of cortical plasticity (Chipchase et al., 2011). For a few years, chronic ISMC has been envisaged as a potential substitute for aerobic physical exercise (EX), i.e., endurance exercise such as walking, running, cycling to improve brain health including mental health. To support this, chronic ISMC was reported to increase the production of myokines involved in neuroplasticity (Sanchis-Gomar et al., 2019) as well as cerebral levels of brain-derived neurotrophic factor (BDNF) (Ke et al., 2011; Dalise et al., 2017) a neurotrophin playing a crucial role in EX-induced synaptic plasticity, neurogenesis and cognition (Vaynman et al., 2004; Lu et al., 2008; Erickson et al., 2012). There are also studies that reported an improvement by chronic ISMC of the negative psychological state associated with spinal cord injury (Twist et al., 1992) and memory impairment in Alzheimer’s patients (Scherder et al., 1995). One unresolved point concerns the mechanisms underlying improved cognitive abilities in response to chronic ISMC, while elucidating these mechanisms is a prerequisite for optimizing protocols of ISMC aimed to improve cognition.

One mechanism underlying the cognitive benefit afforded by either chronic ISMC of a large muscular mass or chronic EX such as running could be the repeated elevation of cerebral blood flow as a result of increased neuronal activity, hypercapnia and increased cardiac output (Willie et al., 2014; Smith and Ainslie, 2017). Each increase of CBF may augment the delivery of myokines involved in neuroplasticity into the brain. In addition, increased blood flow in the cerebral microvasculature may stimulate NO production as a consequence of phosphorylation of endothelial NO synthase (eNOS) at serine 1177 by shear stress (Dimmeler et al., 1999) while NO secreted by the endothelium of cerebral capillaries plays a key role in neurogenesis and neuroplasticity (Chen et al., 2005; Hopper and Garthwaite, 2006). Surprisingly, the effect ISMC of a large muscular mass on regional CBF was never investigated. This effect cannot be extrapolated from studies reporting CBF elevation during acute EX. Indeed, the pattern of neuronal activation differs between voluntary and involuntary contraction (Wegrzyk et al., 2017) and whether neuronal activation occurs in cognition-related brain regions during stimulation as observed during acute EX is not known. In addition, while many studies reported a contribution of cardiac output to CBF (Meng et al., 2015), an independent relationship between cardiac output and CBF was recently questioned (Castle-Kirszbaum et al., 2021).

In this context, the present study aimed to investigate the effect of ISMC of a large muscular mass on regional CBF. For this purpose, CBF was measured at the microvascular level directly by laser doppler flowmetry (LDF_CBF_) and indirectly by changes in tissue p-eNOS^Ser1177^ levels (p-eNOS_CBF_) in anesthetized rats subjected to induced bilateral hindlimb contraction or in unstimulated rats. Selective blockade of ß1 cardiac receptors by atenolol, their activation by dobutamine as well as modulation of stimulation intensity (2.5 vs. 5-fold the motor threshold) were used as strategies to assess the contribution of increased cardiac output to CBF changes. Changes in neuronal activity were assessed from the measurement of c-fos levels. CBF and c-fos levels were measured in the area representing the hindlimb and in two cognition-related brain regions (the hippocampus and the prefrontal cortex). Electrically induced bilateral hindlimb contraction was performed according to an acute stimulation protocol (stimulation for 30 min under ketamine/xylazine or chloral hydrate anesthesia) since an interaction between anesthesia and the effect of ISMC on blood pressure (BP) was previously suspected by Ishide et al. (2002) or to a subchronic stimulation protocol (stimulation of 30 min a day for 7 consecutive days under isoflurane anesthesia).

## Materials and Methods

### Animals and Drugs

Animals. Experiments were conducted according to the French Department of Agriculture guidelines (license 21-CAE-102), approved by local ethics committee (Ethic committee of animal experiment, Dijon, aggregation number 105) and conformed to ARRIVE guidelines. They were carried out on 88 male Wistar rats (7 to 8 week-old) purchased from Janvier Labs (Le Genest Saint Isle, France). Rats were housed under a 12 h/12 h light/dark cycle and allowed free access to food and water.

Anesthetic agents. In experiments for which rats were placed on a stereotaxic frame, volatile anesthesia could not be used. Therefore, electrically induced contraction was first induced in rats anesthetized with ketamine (Virbac, Carros, France)/xylazine (Bayer, Leverkuse, Germany) that is the recommended no volatile anesthesia in rodents. The administration of a mixture (0.115 ml/100 g, i.p) of ketamine (75 mg/kg) and xylazine (8 mg/kg) was preceded (15 min) by the administration of buprenorphine (0.05 mg/kg, s.c, Buprécare, Axience, Pantin, France). An additional injection of ketamine (35 mg/kg) was administrated as indicated by loss of withdrawal reflex to pinching of the hindpaw and/or spontaneous increases in heart rate (HR). Then, anesthesia was induced by chloral hydrate. This anesthetic agent has the reputation to not depress the cardiovascular system even though it is not recommended because of its depressive effect on ventilation when used at the dose required for surgical anesthesia, its irritating effect (peritonitis) when chronically administered by i.p route, its cancerogenic effect when administered chronically. However, in the present study, respiratory depression and toxicity of chloral hydrate could not occur since rats were placed under assisted ventilation and euthanized less than 100 min after induction of anesthesia, respectively. Anesthesia by chloral hydrate consisted in the administration of a 4% chloral hydrate (Sigma-Aldrich, Saint-Quentin Fallavier, France) solution (saline). This solution was administered under a volume of 10 ml/kg (i.p) to induce anesthesia and then (30–40 min later) under a volume of 5 ml/kg (i.v) to maintain deep anesthesia. Pentobarbital anesthesia (60 mg/kg) was used to explore the effect of dobutamine on CBF. For experiments not requiring to place rats on a stereotaxic frame, anesthesia was induced by 4% isoflurane in a clear induction chamber and then maintained with 2% isoflurane in air in a standard rat nose mask. Advantage of gaseous anesthesia is that it allows a rapid awakening as compared to anesthesia with chloral hydrate, thus limiting potential interaction between CBF and anesthesia. Body temperature of anesthetized rats was maintained around 37°C with a heating pad.

Drugs. Atenolol (Sigma-Aldrich, Saint-Quentin Fallavier, France), a water-soluble and specific ß1 receptor competitive antagonist was administered at 10 mg/kg (1 ml/kg, i.v). Dobutamine (Sigma, D0676), a water-soluble specific ß1 receptor agonist was infused at 10 μg/kg/min (5.5 μL/min/100 g for 30 min, i.v). Dosages of atenolol and dobutamine were determined from dosages used in patients to treat tachycardia and low cardiac output hypoperfusion states.

### Electrical Stimulation

Circular (7 mm) electrodes (Contrôle Graphique Medical, Brie-Compte-Robert, France) were connected to an electrical stimulator (DS7AH, Digitimer, Hertfordshire, United Kingdom) controlled by TIDA software (Tida, Heka Elektronik, Lambrecht/Pfalz, Germany) to trigger stimulation. After abdomen and back shaving in anesthetized rats, the anode was placed on the abdomen and the cathode on lumbar (L6) nerve roots to induce the simultaneous contraction of the two hindlimbs. Electrical stimulation was induced with a rectangular biphasic current of 100 Hz frequency, 200 µs of pulse duration with an alternating of 6 s ON (contraction) and 3 s OFF (rest). The current intensity was set to 2.5- or 5- fold the motor threshold (MT) i.e., the smallest stimulation intensity to induce the contraction of both hindlimbs (7 mA). The intensity was continuously increased (up to 70 mA) to maintain the target torque output.

Two protocols of stimulation were used. According to the acute stimulation protocol, ventilated rats anesthetized with ketamine/xylazine or chloral hydrate were subjected to a 30 min-long stimulation period. End-tidal carbon dioxide (CO_2_) was continuously recorded with a capnograph (CapnoScan, Kent Scientific, United States) and maintained at 35 mmHg by adjusting the ventilation rate, thus allowing us to eliminate the effect of hypercapnia on CBF. According to the chronic stimulation protocol, a daily (30 min) stimulation was repeated during 7 consecutive days in rats anesthetized with isoflurane and not placed under assisted ventilation. The rationale for this protocol is that a treadmill activity 30 min a day during 7 consecutive days was reported by our laboratory to increase levels of both c-fos and p-eNOS^Ser1177^ in the sensorimotor cortex, the hippocampus and the prefrontal cortex and to reduce memory deficit induced by scopolamine (Banoujaafar et al., 2014; Cefis et al., 2019; Pedard et al., 2019). The parameters of stimulation did not differ between the acute and chronic protocols of stimulation.

### Methods to Investigate Cerebral Blood Flow

Changes in CBF during stimulation were assessed at the microvascular level as interrogation of the cerebral microvasculature provides a more accurate assessment of actual tissue perfusion than macrovascular hemodynamic measurements. Thus, CBF was directly assessed from laser doppler flowmetry (LDF_CBF_) and indirectly from changes in tissue p-eNOS^Ser1177^ levels (p-eNOS_CBF_). LDF method measures red blood cells velocity in the cerebral microcirculation while changes in cerebral p-eNOS^Ser1177^ levels reflect changes in shear stress in vessels of the cerebral microcirculation mainly the capillaries. Supporting changes in p-eNOS^Ser1177^ levels as a reliable marker of changes in CBF at the microvascular level, cerebral peNOS^Ser1177^ levels decreased in response to interruption of the carotid circulation (Banoujaafar et al., 2016) and increased in response to EX in the sensory-motor cortex, the prefrontal cortex and the hippocampus (Cefis et al., 2019; Pedard et al., 2019). Notably, even though eNOS phosphorylation is induced by both flow-and receptor-dependent mechanisms, an increase in microvasculature flow obligatorily translates into the induction of eNOS phosphorylation at serine 1177.

### Laser Doppler Flowmetry

LDF_CBF_ was expressed as arbitrary tissue perfusion units (TPU) and continuously recorded before, during and after acute stimulation using a probe (diameter of 1.2 mm) connected to a laser Doppler flowmetry apparatus (BLF21, Transonics Systems, NY, United States). After anesthesia (with chloral hydrate or pentobarbital) and heparin administration (50 UI/100 g, i.v, Lovenox, Aventis, Strasbourg, France), arterial BP and heart rate (HR) were recorded from a catheter inserted into a common carotid artery, the caudal artery or a femoral artery using a BP monitor (Easy Graf, GOULD, United States). Rats were then ventilated (Harvard Apparatus, Fircroft, Eddenbridge, United Kingdom) with room air through an endotracheal tube and placed on a stereotaxic frame (Model 900, Kopf Instruments, Tujunga, CA, United States). The tip of the probe was placed perpendicularly to the cortical surface and centered on the area representing the hindlimb according to the following stereotaxic coordinates: AP = −1.8 mm, L = 2.8 mm from bregma as reference (Atlas of Paxinos and Watson). For this purpose, a circular area of 5 mm diameter overlying the cortical hindlimb representation was thinned using a dental drill until a translucent cranial plate remained. Then, this plate was removed before positioning the probe on the dura mater. Placement of the probe on area with large vessels was avoided. Rats with a mean arterial BP (MABP) below 60 mmHg were excluded for further experiments. LDF_CBF_ was recorded in rats stimulated at low (2.5x MT) or high intensity (5x MT), in rats with or without treatment with atenolol as well as in unstimulated rats receiving dobutamine.

### Western Blotting Analysis

The cortical area representing the hindlimb, the hippocampus and the prefrontal cortex were collected 20 min after cessation of stimulation in rats subjected to the acute stimulation protocol, 24 h after the last session of stimulation in rats subjected to the sub-chronic stimulation protocol and at equivalent times in corresponding unstimulated sham rats. Once collected, the structures were immediately weighed and frozen at -80°C. Levels of p-eNOS^Ser1177^ as an indirect marker of CBF (p-eNOS_CBF_) and of c-fos as a marker of neuronal activation were measured by Western blotting analysis using Stain-Free imaging technology (Biorad). Briefly, equal amounts of protein were loaded on sodium dodecyl sulfate–polyacrylamide (SDS-PAGE) TGX Stain-Free FastCast gel electrophoresis (TGX Stain-Free FastCast Acrylamide Kit, 7.5%, 1610181, Bio-Rad) and electrophoretically transferred to polyvinylidene difluoride (PVDF) membranes using Turbo Transblot technology (1704150, Biorad). After blocking non-specific binding sites for 1 h at room temperature (RT), membranes were incubated overnight at 4°C with primary antibody directed against p-eNOS^Ser1177^ (1/1000, mouse monoclonal, 612,383, BD Biosciences) or c-fos (1/3,000, rabbit polyclonal, GTX129846, GeneTex). Membranes were then incubated (1 h, RT) with secondary antibody conjugated with horseradish peroxidase (1/25000, anti-mouse: 115-035-166, anti-rabbit: 111-035-144, Jackson ImmunoResearch). Membranes were then placed in Chemidoc imaging systems. A stain-free image of the blot was captured to control the total protein loading and normalize data. Protein-antibody complexes were visualized using the enhanced chemiluminescence Western blotting detection system (ECL 2, 1151-7371, Fisher Scientific). Band densities were analysed with ImageLab software (Bio-Rad) and standardized on total protein. Gels were run in duplicate. The appropriate amounts of total proteins to be analysed were previously determined from concentration (increasing amounts of proteins)/response (optical density of the band) curves.

### Experimental Design

Four sets of experiments were conducted. The first set aimed to assess the peripheral cardiovascular effect (HR and BP recorded from a catheter inserted in a common carotid artery) of acute stimulation in rats anesthetized with either ketamine/xylazine (*n* = 12) or chloral hydrate (*n* = 6). The second set of experiments investigated the effect of acute stimulation on CBF in the area representing the hindlimb, the hippocampus and the prefrontal cortex in rats anesthetized with chloral hydrate (*n* = 31). In the same stimulated rats, LDF_CBF_ was measured in the area representing the hindlimb while p-eNOS_CBF_ was measured in the three brain regions of interest. HR and BP were recorded from a catheter inserted either in a common carotid artery (*n* = 13) or in the caudal artery (*n* = 12) in order to exclude a potential interaction between CBF and unilateral carotid occlusion. The third set of experiments investigated the contribution of cardiac output to the CBF response to acute stimulation (*n* = 29). It consisted in the recording of HR, BP (recorded from a common carotid artery, a caudal artery, or a femoral artery) and LDF_CBF_ in the area representing the hindlimb either in stimulated rats treated with a β-blocker (*n* = 12), in rats stimulated at a lower intensity (*n* = 5) than that used in the previous experiments or in unstimulated rats treated with dobutamine (*n* = 12). The aim of the fourth set of experiments (*n* = 10) was to investigate the effects of subchronic stimulation on levels of c-fos and p-eNOS^Ser1177^ in the three regions of interest in isoflurane-anesthetized rats (5 stimulated rats and 5 sham rats).

### Statistical Analysis

Values are expressed as mean ± standard deviation (SD). The normality of the values was carried out by a Shapiro-Wilk test. To study the effect of stimulation on HR, MABP and CBF over time, a one-way or two-way ANOVA (time x group) was performed with time 0 or −10 min as reference time (control values). *p* values were subjected to a Bonferroni correction. Differences in p-eNOS^Ser1177^ and c-fos levels between groups of rats were assessed using unpaired *t*-test. Statistical significance was set at the 5% level.

## Results

### Preliminary Experiments to Indirectly Assess the Effect of Stimulation on Cardiac Output

**FIGURE 1:**
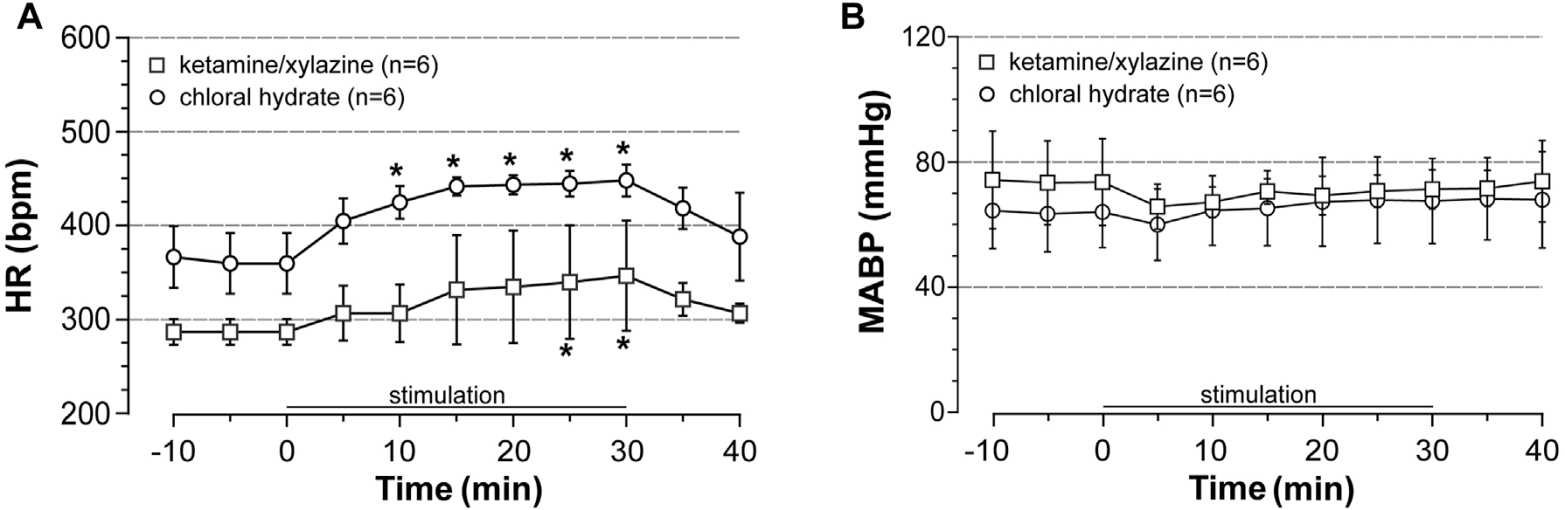
Effects of induced contraction on heart rate (HR) and blood pressure (BP). HR and mean arterial blood pressure (MABP) were recorded before, during (0–30 min) and after electrically induced contraction of hindlimbs (5x MT) in rats anesthetized either with ketamine/xylazine or chloral hydrate. Values are expressed as means ± SD, *n* = number of rats. ^*^ different from pre-stimulation values at *p* < 0.01 and no difference between anesthetic agents after two-way (time x group) ANOVA and Bonferroni correction.

Increased cardiac output was reported to contribute to the elevation of flow in the cerebral arteries that occurs during acute EX involving a large body mass (Seifert et al., 2009). Therefore, to investigate whether cardiac output increased during our protocol of stimulation, we measured the response of BP and HR to acute stimulation protocol (intensity at 5-fold motor threshold). Experiments were first conducted in ventilated rats anesthetized with ketamine/xylazine (*n* = 12), BP and HR being recorded from a catheter inserted into the right common carotid. Among 12 ketamine/xylazine-anesthetized rats, half died before the end of stimulation likely as a result of cardiovascular arrest. In the surviving rats, stimulation induced a time-dependent increase in HR but did not change BP (Figure 1). Tachycardia at 30 min of stimulation reached + 25% of the pre-stimulation values and the HR rapidly recovered pre-stimulation values after stopping the stimulation. From these data, increased cardiac output was expected to occur during stimulation. However, the substantial mortality (50%) observed in rats anesthetized with ketamine/xylazine in combination with the suspicion of interaction between anesthesia and the effect of ISMC on BP (Ishide et al., 2002) led us to conduct the same experiments in artificially ventilated rats anesthetized with choral hydrate. As observed under ketamine/xylazine anesthesia, stimulation at 5-fold motor threshold induced a time-dependent elevation of HR without associated changes in BP (Figure 1) in chloral hydrate-anesthetized rats (*n* = 6). The superimposable response of HR and BP to stimulation between rats anesthetized with ketamine/xylazine and those anesthetized with chloral hydrate indicated that stress (adrenalin secretion)- if occurs during stimulation- did not differ between the two protocols of anesthesia. Furthermore, unlike ketamine/xylazine, chloral hydrate did not induce animal death. Therefore, chloral hydrate was used in the further experiments on rats subjected to acute stimulation protocol.

### Effect of Induced Contraction on LDF_CBF_ in the Area Representing the Hindlimb

In the preceding experiment, BP was recorded from a catheter inserted into the common carotid artery due to the ability to easily insert a catheter into this large vessel and of the obtention of a large pulsatile pressure from which HR can be estimated without any difficulty. However, vascular catheterization resulted in a permanent occlusion of the vessel that may interact with the response of CBF to stimulation. Indeed, acute common carotid artery occlusion was reported to reduce basal global CBF (De Ley et al., 1985). Therefore, the effect of the acute stimulation protocol on local CBF was investigated in rats in which HR and BP were recorded from either the left common carotid artery (carotid series) or the caudal artery (caudal series). The probe was placed on the right cortical area representing the hindlimb. This region and the two cognition-related brain regions were then collected for further determinations of p-eNOS^Ser1177^ levels. Notably, CBF was not measured in six rats (on 31) as their MABP dropped below 60 mmHg during LDF measure. As shown below, no statistical difference was observed between the two series indicating that unilateral carotid occlusion did not interact with the response of LDF_CBF_ to electrically induced contraction.

**FIGURE 2:**
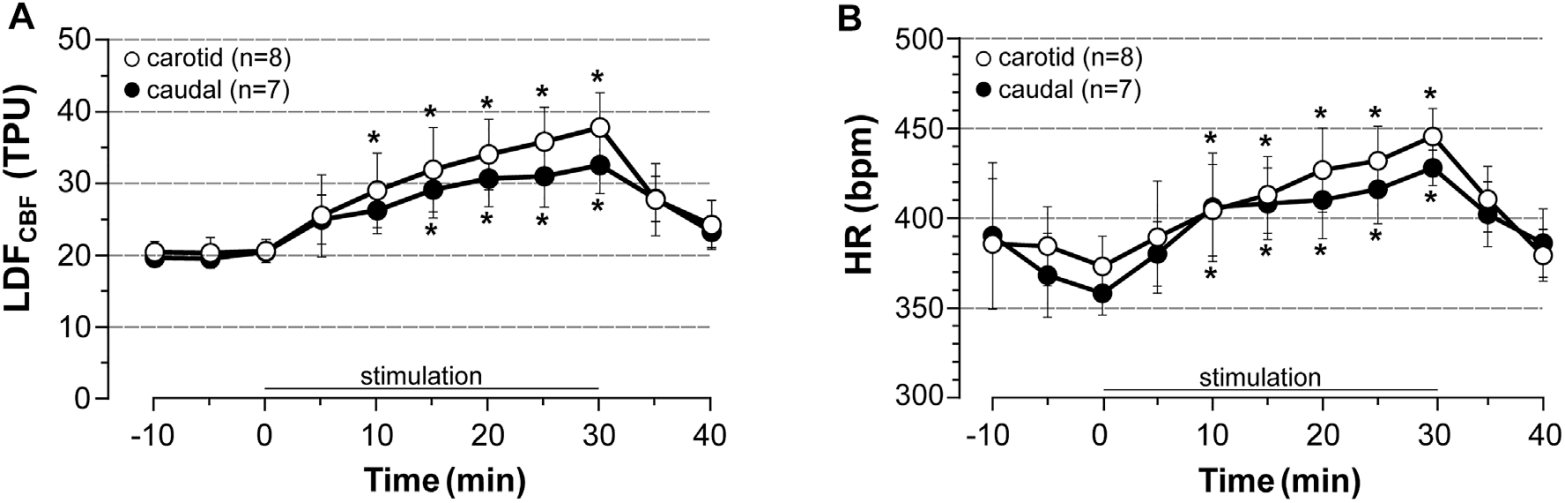
Effects of induced contraction on LDF_CBF_ in the area representing the hindlimb LDF_CBF_ in the cortical area representing the hindlimb and HR were measured before, during (0–30 min) and after electrically induced contraction of hindlimbs (5x MT). HR was calculated from a BP trace that was recorded from the common carotid artery (carotid series) or the caudal artery (caudal series) in rats anesthetized with chloral hydrate. Values are expressed as means ± SD, *n* = number of rats. ^*^ significantly different from pre-stimulation values at *p* < 0.01 and no difference between series after two-way (time x group) ANOVA and Bonferroni correction.

In unstimulated sham rats of both series (*n* = 5 rats each), a slight (less than 7% of control values) and unsignificant decrease in HR, MABP and CBF were observed over time (not shown). By contrast, induced contraction (5x MT) evoked a time-dependent elevation in LDF_CBF_ and HR that rapidly returned to pre-stimulation values after cessation of stimulation (Figure 2). Thus, at 30 min of stimulation and as compared to pre-stimulation values, CBF was significantly increased by 84% in rats of the carotid series (*n* = 8) and 65% in rats of the caudal series (*n* = 7) (Figure 2A, with no significant difference between the two series). Moreover, as previously observed (see results in 3.1) stimulation increased HR (+ 25% at 30 min of stimulation) in both series without differences between the series (Figure 2B) and did not change MABP (not shown). No statistical difference in HR was observed between carotid and caudal series.

### Effect of Induced Contraction on p-eNOS_CBF_ in the Area Representing the Hindlimb, the Hippocampus and the Prefrontal Cortex

**FIGURE 3:**
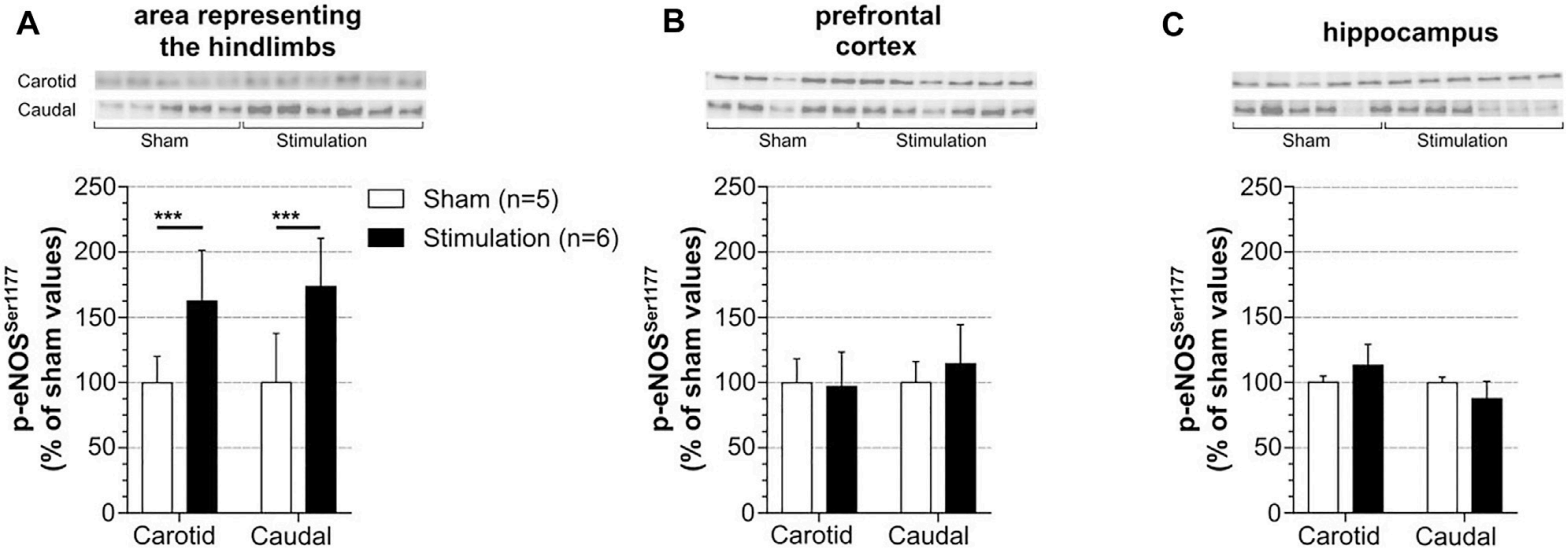
Effect of induced contraction on p-eNOS_CBF_ levels of p-eNOS^Ser1177^ were measured in the sensorimotor cortex **(A)**, prefrontal cortex **(B)** and hippocampus **(C)** 20 min after cessation of a 30 min electrically induced contraction of hindlimbs (5x MT) in rats with catheterization of the common carotid artery (carotid series) or the caudal artery (caudal series). These rats are the same than those used to measure LDF_CBF_. Values are expressed as means ± SD, *n* = number of rats. ^***^ significantly different from sham rat values after *t*-test at *p* < 0.001.

Regarding the experimental difficulty to simultaneously measure CBF in different brain regions with LDF and the lack of validation of LDF method to explore subcortical CBF, changes of p-eNOS^Ser1177^ expression were used as an indirect marker of changes in CBF. Levels of p-eNOS^Ser1177^ were measured in the regions of interest in rats in which LDF_CBF_ was recorded (see paragraph 3.2). The brains were collected 20 min after cessation of stimulation (at 5x MT) in stimulated rats or at the equivalent time in unstimulated sham rats. The results are summarized in Figure 3. Supplemental Figure S1 shows full membranes as well as loading controls (stain-free) from which graphs of Figure 3 were constructed. In accordance with elevation of LDF_CBF_ in the area representing the hindlimb (Figure 2), this region exhibited increased p-eNOS^Ser1177^ levels in response to stimulation. Thus, as shown in Figure 3A, the rise in p-eNOS^Ser1177^ expression in the area representing the hindlimb reached +63% in the carotid series and +69% in the caudal series (*p* < 0.001), as compared to values obtained in unstimulated sham rats. By contrast, p-eNOS^Ser1177^ levels in the prefrontal cortex (Figure 3B) and the hippocampus (Figure 3C) did not differ between stimulated and unstimulated sham rats suggesting that CBF did not increase in these cognition-related regions.

### Contribution of Cardiac Output to LDF_CBF_

**FIGURE 4:**
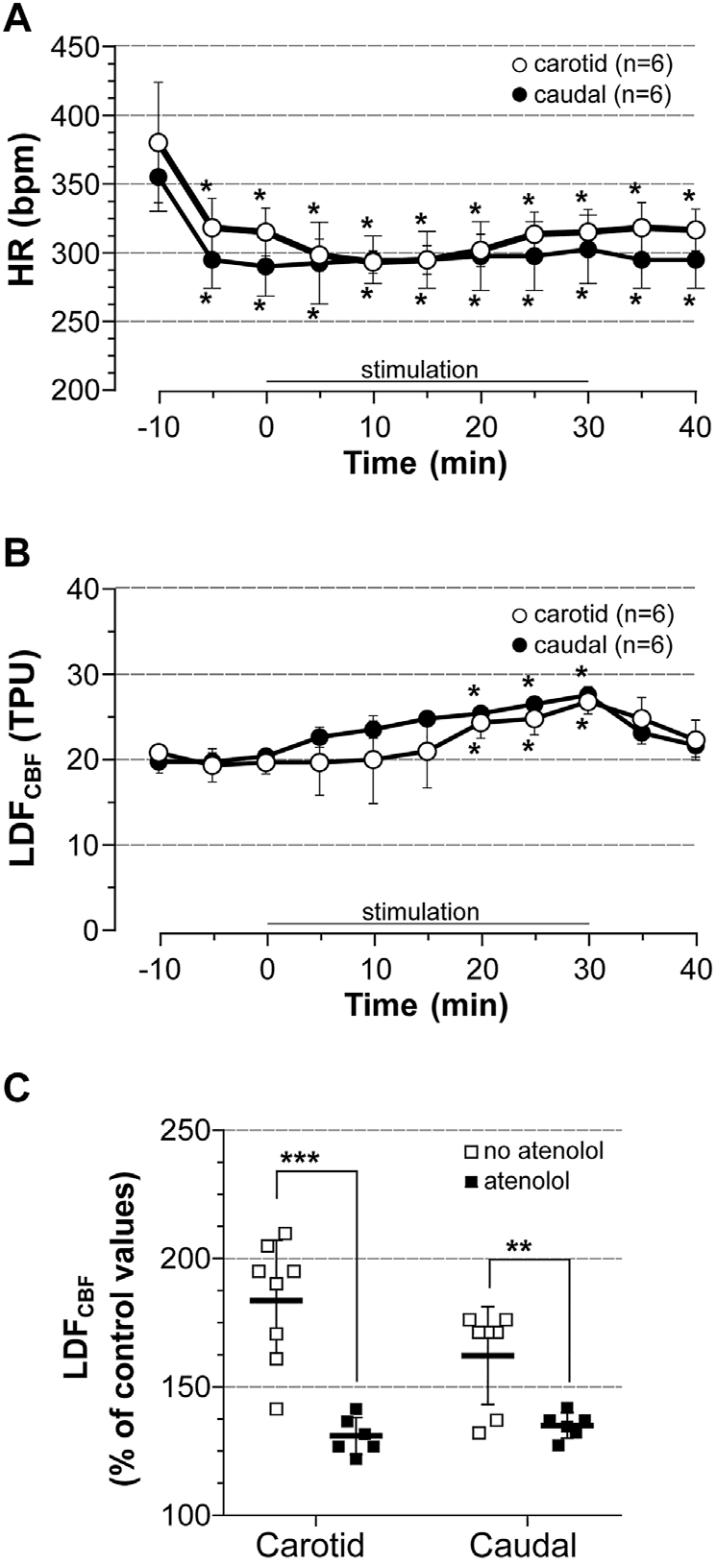
Cardiovascular effect of induced contraction in atenolol-treated rats. Atenolol (10 mg/kg, i.v.) was administrated 10 min before (time -10) bilateral hindlimb contraction at 5x motor threshold. **(A)** HR was calculated from BP trace that was recorded from the common carotid artery (carotid series) or the caudal artery (caudal series), **(B)** LDF_CBF_ was measured in the cortical area representing the hindlimb, **(C)** Difference in CBF expressed as % of control values at 30 min of stimulation in rats treated with atenolol (full squares) *vs*. untreated rats (empty squares). Values are expressed as means ± SD, *n* = number of rats. ^*^ significantly different from pre-stimulation values at *p* < 0.01 and no difference between series after two-way (time x group) ANOVA and Bonferroni correction.

The contribution of cardiac output to the elevation of LDF_CBF_ in the area representing the hindlimb in response to stimulation was first investigated by the measurement of LDF_CBF_ in stimulated rats treated with the β-blocker atenolol. Atenolol was administrated 10 min before the onset of stimulation at 5 x MT, HR and BP being recorded either from the left common carotid (carotid series, *n* = 6) or the caudal artery (caudal series, *n* = 6) with the LDF probe placed on the right area representing the hindlimb. Results are summarized in Figure 4. Atenolol fully prevented the chronotropic effect of stimulation either in the carotid series or in the caudal series (Figure 4A, note the bradycardia induced by atenolol before induction of stimulation). By contrast, stimulation still induced a time-dependent increase in LDF_CBF_ (Figure 4B) but the increase was significantly lower than that observed in untreated rats (Figure 4C). Thus, CBF values at 30 min of stimulation were 26.8 and 27.6 TPU in the carotid and caudal series, respectively (i.e., 131 and 135% of control values). BP remained unchanged before and after stimulation in atenolol-treated rats (not shown).

**FIGURE 5:**
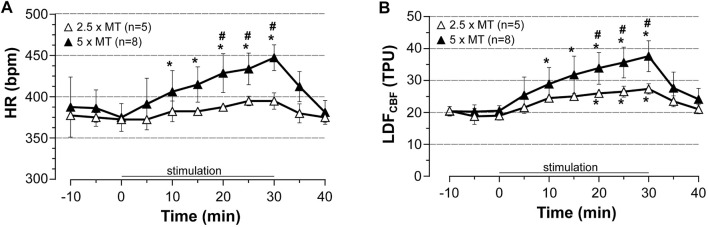
Influence of stimulation intensity of induced contraction-associated cardiovascular effects. HR and LDF_CBF_ in the cortical area representing the hindlimb were recorded before, during (0–30 min) and after electrically induced contraction of hindlimbs at 2.5 or 5x motor threshold. Values are expressed as means ± SD, *n* = number of rats. ^*^ significantly different from pre-stimulation values at *p* < 0.01, ^#^ significant difference between low and high intensity after two-way (time x group) ANOVA and Bonferroni correction.

Then, the cardiac response to stimulation was modulated by changing intensity of stimulation. Thus, LDF_CBF_ in the area representing the hindlimb, HR and BP (recorded from common carotid artery only) were measured before, during, and after stimulation at 2.5x MT in chloral hydrate anesthetized rats (*n* = 5) and compared to those obtained after stimulation at high (5x MT) intensity**.** As shown in Figure 5, stimulation at low intensity increased HR only by 10% (NS) and CBF by 44% (*p* = 0.0251) at 30 min of stimulation as compared to pre-stimulation values. These changes were significantly lower than those evoked by high-intensity stimulation, at least from 20 to 30 min of stimulation but significantly higher (*p* = 0.0317) than those observed in corresponding sham rats (not shown). Of note, MABP remained to pre-stimulation values during stimulation at low intensity (not shown).

**FIGURE 6:**
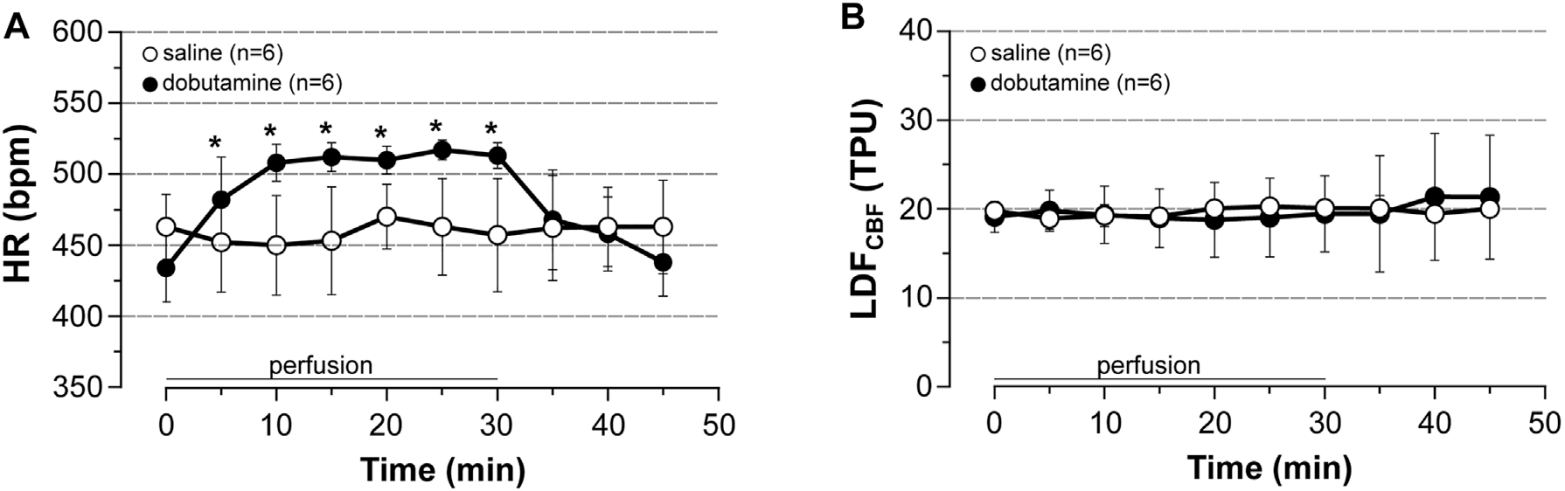
Cardiovascular effects of dobutamine. Dobutamine (10 μg/kg/min, 5.5 μl/min/100 g for 30 min, i.v) or saline was perfused to unstimulated rats. **(A)** HR was calculated from BP trace that was recorded from the femoral artery, **(B)** LDF_CBF_ was measured in the cortical area representing the hindlimb. Values are expressed as means ± SD, *n* = number of rats. * significantly different from control values after one-way ANOVA and Bonferroni correction at *p* < 0.05.

Lastly, we investigated the effect of dobutamine (an agonist of β1-receptor able to increase cardiac output but unable to alter neuronal activity as a result to its incapacity to cross the blood-brain barrier) in unstimulated rats. LDF_CBF_ was continuously recorded in the cortical area representing the hindlimb, BP and HR being measured from a catheter inserted into a femoral artery in rats anesthetized with pentobarbital. As shown in Figure 6 and as compared to saline, dobutamine increased HR by 18% (+80 beats/min) after 30 min of perfusion (Figure 6A), while it changed neither LDF_CBF_ (Figure 6B) nor MABP (not shown).

### Cerebral Effects of Subchronic Stimulation

**FIGURE 7:**
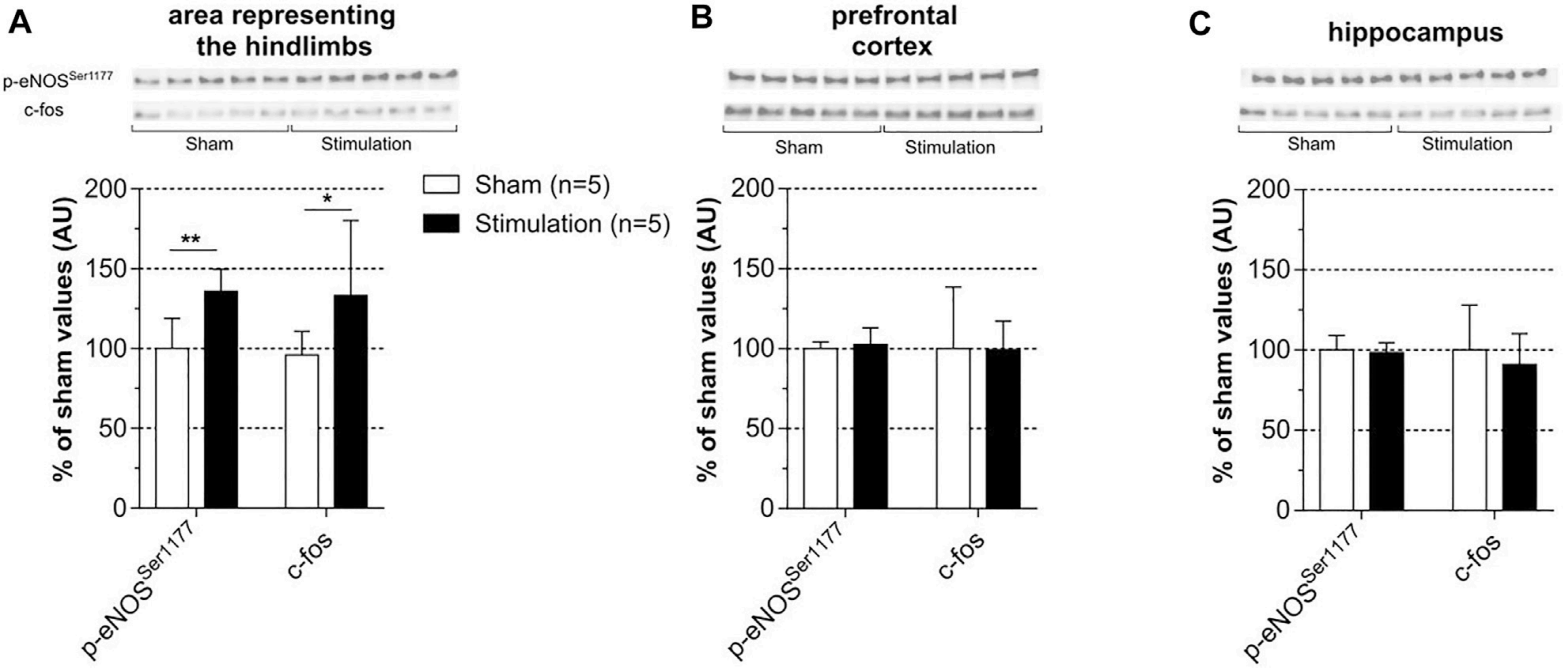
Effect of subchronic stimulation on p-eNOS^Ser1177^ and c-fos brain levels. Levels of c-fos and p-eNOS^Ser1177^ were measured in the area representing the hindlimb **(A)**, prefrontal cortex **(B)** and hippocampus **(C)** 24 h after the last session of a 30 min electrically induced contraction of hindlimbs (5x MT) repeated during 7 consecutive days in rats anesthetized with isoflurane and in sham unstimulated. Values are expressed as means ± SD, *n* = number of rats. * (*p* < 0.05) and ** (*p* < 0.01) significantly different from sham rat values after *t*-test.

Subchronic stimulation was used to investigate the effect of stimulation of c-fos as a marker of neuronal activation. Levels of c-fos and p-eNOS^Ser1177^ were measured in the three regions of interest that were collected 24 h after the last session of stimulation. In fact, c-fos was not investigated in rats subjected to the acute stimulation protocol because -in these rats- the brain regions were collected 20 min after cessation of stimulation, a time too short to reveal potential c-fos protein upregulation. As shown in Figure 7 and as compared to unstimulated sham rats, induced contraction increased c-fos and p-eNOS^Ser1177^ levels in the cortical area representing the hindlimb (Figure 7A) with no change in the prefrontal cortex (Figure 7B) and the hippocampus (Figure 7C). Supplemental Figure S2 shows full membranes as well as loading controls (stain-free) from which graphs of Figure 7 were constructed. Importantly, the selective increase in p-eNOS^Ser1177^ in the area representing the hindlimb suggests that hypercapnia did not occur during stimulation. Consistently, spontaneous hyperventilation was noticed during each period of stimulation.

## Discussion

The main results provided by the present study are that 1) stimulation increased LDF_CBF_ and p-eNOS^Ser1177^ levels in the area representing the stimulated muscles but did not change p-eNOS^Ser1177^ levels in the prefrontal cortex and the hippocampus, 2) LDF_CBF_ elevation was dependent on the intensity of stimulation, blunted by atenolol administration and not reproduced by dobutamine administration in unstimulated rats, 3) stimulation resulted in a selective augmentation in c-fos levels in the area representing the stimulated muscles.

In opposition to the plethora of studies on the cardiovascular (CV) effects of physical activity, the CV effects of ISMC are poorly documented. Nevertheless, tachycardia was consistently reported during induced contraction (Crayton et al., 1979; Ishide et al., 2002). More controversial and for still hypothetical reasons (Ishide et al., 2002) is the effect of ISMC on arterial BP (an augmentation, a decrease or no effect), while BP increases during physical activity as a consequence of increased cardiac output. In the present study, tachycardia increased by 25% after 30 min of stimulation while BP remained to pre-stimulation values irrespective of the anesthetic agent (ketamine/xylazine or chloral hydrate). Of note, tachycardia without an associated change in BP was previously reported during bilateral sciatic nerve stimulation in anesthetized rats (Ishide et al., 2002). Tachycardia during stimulation related to activation of bulbar sympathetic centers and subsequent increased sympathetic outflow as evidenced from the complete prevention of tachycardia by atenolol. Notably, while sympathetic activation observed during voluntary contraction is driven by the central command (feed-forward mechanism originating from higher brain centers) (Williamson et al., 2006), the exercise pressor reflex (a feed-back mechanism originating from skeletal muscle) and the arterial baroreflex (a negative feed-back mechanism originating from the carotid sinus and aortic arch) (Fisher et al., 2015), it is driven only by the exercise pressor reflex during our protocol of involuntary contraction since the central command is absent during stimulated contraction and BP did not change during stimulation. Thus, only the activation of sensory fibers originating from muscles may be responsible for the sympathetic activation observed during stimulation. According to our protocol, activation of these fibers was due not only to muscle contraction but also to electrical stimulation of the dorsal roots (that contain sensory fibers).

To the best of our knowledge, our study is the first to investigate the effect of involuntary contraction involving a large muscular mass on regional CBF. CBF was directly and indirectly investigated by LDF measures (LDF_CBF_) and changes in p-eNOS^Ser1177^ levels (p-eNOS_CBF_), respectively. Our results showed a region-dependent increase in CBF during electrically induced bilateral hindlimb contraction. More precisely, LDF_CBF_ and p-eNOS_CBF_ increased in the area representing the hindlimb, while p-eNOS_CBF_ did not change in cognition-related brain regions in rats subjected to acute stimulation. The mechanisms that control blood supply to the brain are complex and multiple. However, both local factor i.e neuronal activity and systemic factor i.e BP, arterial partial pressure of CO_2_ and cardiac output are involved (Willie et al., 2014). In the present study, LDF_CBF_ in the cortical area representing the hindlimb related neither to hypercapnia nor change in BP as end-tidal CO_2_ pressure was maintained at 35 mmHg during stimulation by increasing the ventilation rate and BP remained to pre-stimulation values during stimulation. By contrast, our results on atenolol and stimulation intensity support the contribution of increased cardiac output to LDF_CBF_ elevation. However, despite the elevation of cardiac output evoked by stimulation, p-eNOS_CBF_ was not increased in all the regions examined indicating that increased cardiac output cannot by itself induce CBF elevation. Consistently, dobutamine failed to reproduce the effect of stimulation on LDF_CBF_. Of note, pharmacologically induced cardiac output and MABP elevation by dobutamine was previously reported to not change global CBF in monkeys (Bandres et al., 1992) while this strategy was recently reported to increase blood flow in the external carotid artery and reduce flow in the internal carotid artery in human beings (Ogoh et al., 2017). Such redistribution of cardiac output to extra-cranial structures likely emphasizes the importance of preventing brain overperfusion. In other words, increased cardiac output observed during stimulation cannot increase CBF in regions where neuronal activation is absent. This is confirmed by our results on c-fos that revealed a selective increase in neuronal activity in the area representing the hindlimb after chronic stimulation and pointed the importance of the voluntary nature of the contraction to expect increased neuronal activity in the cognition-related brain regions. The absence of c-fos elevation in the cognition-related brain regions is in line with a recent study showing no change in hippocampal c-fos in mice subjected to bilateral hindlimb contraction twice a week during 7 weeks (Gardner et al., 2020). Our results showing that increased levels of c-fos coexisted with increased levels p-eNOS^Ser1177^ confirm that it is neuronal activation that drives CBF elevation at the microvascular level during stimulation and argue for a contribution of increased cardiac output to CBF elevation only in the region where increased neuronal activity is present. Notably, EX (treadmill activity, 30 min a day for 7 consecutive days) was reported by our laboratory to induce c-fos upregulation and phosphorylation of eNOS at serine 1177 not only in the sensorimotor cortex but also in the hippocampus and the prefrontal cortex (Banoujaafar et al., 2014; Cefis et al., 2019; Pedard et al., 2019). These data are in line with studies that reported increased CBF (Nishijima et al., 2012) and neuronal activation (Nguyen et al., 2004) in cognition-related brain regions during EX. Such difference between induced and voluntary contraction indicates that muscle contraction alone cannot fully reproduce the effect of EX on cognition-related brain regions. Moreover, increased neuronal activity in the area representing the hindlimbs is likely lower during stimulation than during EX at least in rats. Indeed, assuming that motor-sensory overlap is important for the hindlimb representation in rats (Hummelsheim and Wiesendanger, 1985), neuronal activation in this region involves both the voluntary motor command and activation of sensory fibers originating from active muscles during EX as expected from the projection on the somatosensitive cortex of proprioceptive information (Landgren and Silfvenius, 1969; Wiesendanger and Miles, 1982) but only the latter during stimulation. Supporting this, changes in p-eNOS^Ser1177^ and c-fos levels in the area representing the hindlimb were higher after EX than after subchronic stimulation. Of note, it is unlikely that anesthesia was a confounding factor in the interpretation of our results since neither chloral hydrate nor isoflurane reduced basal CBF as compared to conscious rats (Suzuki et al., 2021).

Limitations of the study. A limitation of the present study is that CBF changes in cognition-related brain regions were indirectly assessed from changes in p-eNOS^ser1177^.This method is less accurate than LDF_CBF_ method for two reasons. The first reason is that it requires to compare p-eNOS^Ser1177^ levels in a group of sham unstimulated rats *vs*. a group of stimulated rats, while by contrast LDF_CBF_ is measured before and after stimulation in the same rats. However, as shown in supplemental figures, the interindividual variability in p-eNOS^Ser1177^ levels in unstimulated rats is low. The second reason is that p-eNOS^Ser1177^ levels were measured by Western blotting analysis that is a semi quantitative method. Another limitation of the present study is the lack of demonstration that increased CBF in the area representing the hindlimb was effectively driven by increased neuronal activity even though increased CBF was observed only in regions with increased neuronal activity. Nevertheless, the possibility that increased CBF in the area representing the stimulated muscles involved the activation of the intracerebral vasodilating cholinergic nerve fibers originating from the basal forebrain (Sato et al., 2001) cannot be excluded. However, evidence that activation of the basal forebrain observed during mastication muscle activity was induced by the cerebral command from the motor cortex, independently of feedback from contracting muscles (Hotta et al., 2020) does not argue for the occurrence of basal forebrain activation during hindlimb stimulation.

We concluded that stimulated contraction of a large muscle mass increases CBF in the area representing the stimulated muscles but not in cognition-related regions and that this selective CBF increase involves an increase in both neuronal activity and cardiac output. The absence of CBF changes in cognition-related brain regions does not support the involvement of flow-dependent neuroplasticity (cerebral hemodynamics) in the pro-cognitive effect of electrically induced contraction. From a physiological point of view, our results provide evidence that local CBF does not increase in response to increased cardiac output alone (without changes in BP and arterial pCO_2_) at least in regions where metabolic needs are not increased. Importantly, during the review processing of the present study was published a work that explored the effect of electrical myostimulation on large muscle mass on CBF in normal human beings using a color-coded ultrasound system (Ando et al., 2021). The authors reported increased flow in the internal carotid artery (+12%), but no change in the vertebral artery. Regarding the association observed between CBF and pCO_2_ changes for the carotid, but not the vertebral circulation, the differential effect of stimulation between the two circulations was related to the difference in the cerebrovascular response to hypercapnia. However, the mechanisms underlying increased flow in the internal carotid artery were not investigated. Our results support the involvement of an increase in both neuronal activity and cardiac output.

## Data Availability

The original contributions presented in the study are included in the article/Supplementary Material, further inquiries can be directed to the corresponding author.
