# Supplementary figures and images for: Region-Dependent Increase of Cerebral Blood Flow During Electrically Induced Contraction of the Hindlimbs in Rats
Source: Front Physiol. 2022 Mar 23;13:811118. doi: 10.3389/fphys.2022.811118 (PMC9040888; doi:10.3389/fphys.2022.811118)

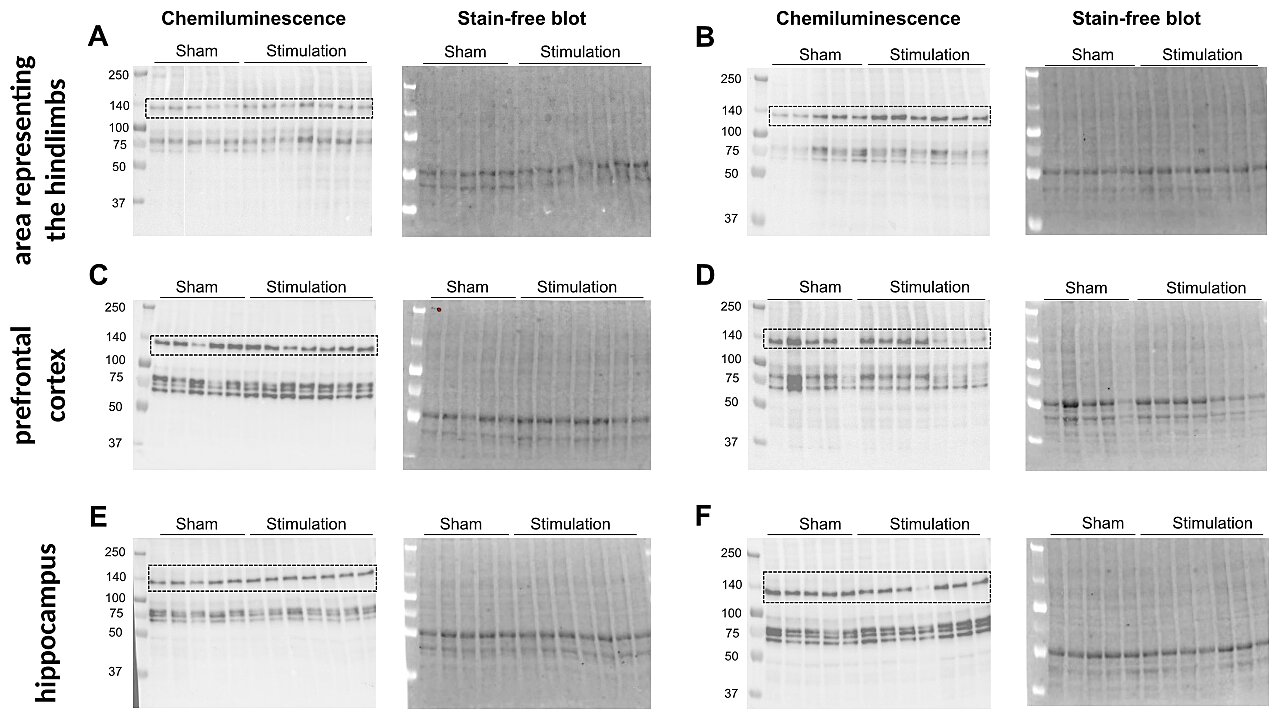

Supplement: Supplementary file 1 [file Image1.JPEG]

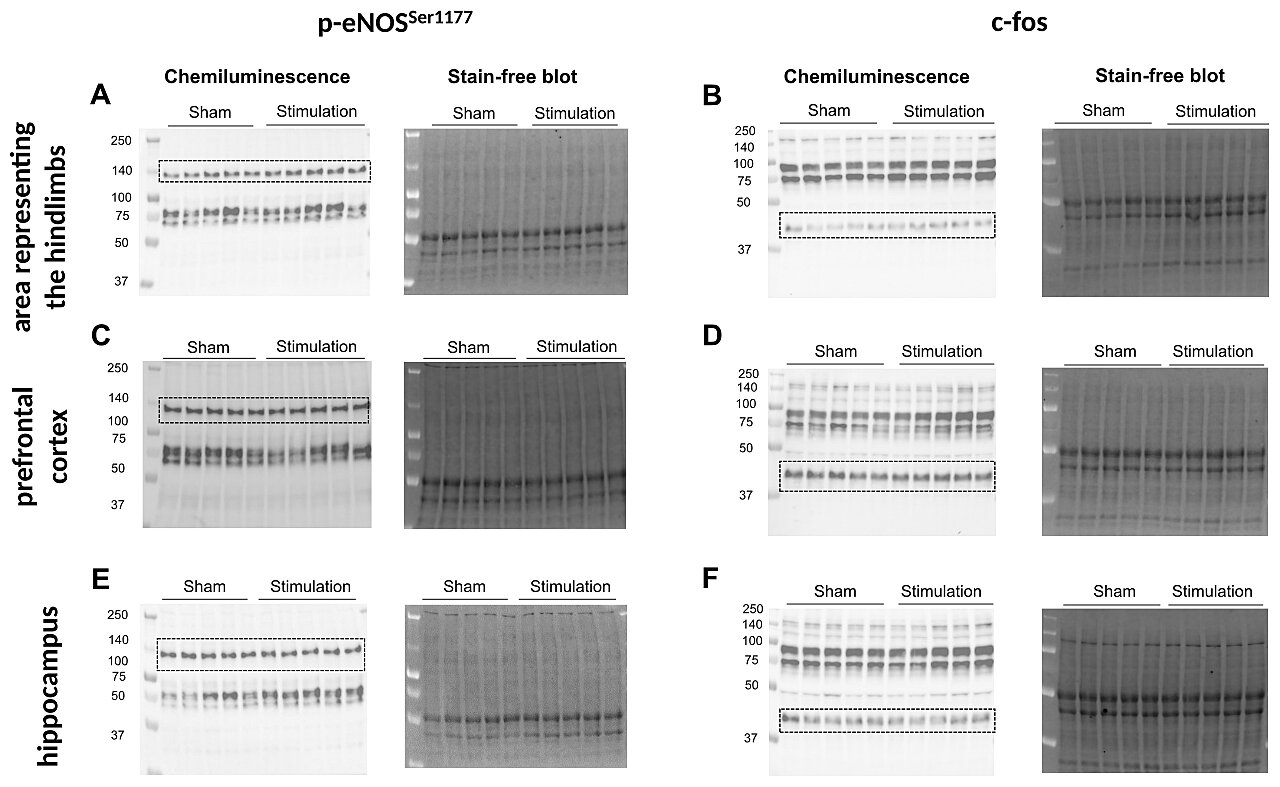

Supplement: Supplementary file 2 [file Image2.JPEG]
